# Various Reduced Graphene Oxide Green Synthetic Routes: Comparing the Cost Procedures

**DOI:** 10.1021/acsomega.5c04090

**Published:** 2025-08-04

**Authors:** Despina A. Gkika, Konstantinos N. Maroulas, George Z. Kyzas

**Affiliations:** Hephaestus Laboratory, School of Chemistry, Faculty of Sciences, 37791 Democritus University of Thrace, Kavala GR-65404, Greece

## Abstract

Biogenic or green-reduced graphene derivatives have emerged and found applications across diverse domains, including the detection of volatile organic compounds, biomedical uses, stretchable electronics, energy storage, photodetectors, high-contrast displays, and optoelectronic and photochemical technologies. These also encompass areas such as the degradation of waterborne pollutants and electrochemical systems like supercapacitors, lithium-ion storage devices, and various sensing applications. The synthesis of graphene and its derivatives (usually graphene oxide (GO)) frequently involves expensive and hazardous chemicals, posing risks to both the environment and human health. However, advancements in polymer composite research have increasingly fostered interdisciplinary collaboration among scientists, steering the field toward more sustainable practices. Previous empirical studies point out that the rising cost of synthesis is becoming an unavoidable factor in the design of new synthetic methodologies, although cost data is often scarce. One approach for the reduction of expenses is to streamline and optimize the synthesis process, such as by simplifying synthetic routes. The suitability of activity-based cost data for low-cost synthesis decisions, specifically for reduced graphene oxide (rGO), was assessed. Common methods involved complex, multistep procedures with cumbersome preparation phases. First, the synthesis is optimized by introducing guarana as a greener reducing agent. To further analyze cost and green considerations, the number of steps in the process was reduced from eight to three, resulting in time and cost savings. The most remarkable aspect of this new synthetic route is its simultaneous application of green chemistry principles and activity-based costing, which improves both the yield and sustainability of the key steps. Economically, compared to the common method costing 248.64 €/g (with eight steps), this streamlined approach cost 19.48 €/g (with three steps), mainly due to reductions in chemical and energy usage.

## Graphical abstract

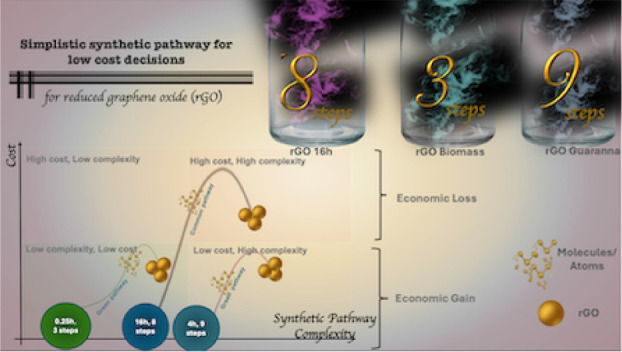

## 1 Introduction

The sequence of steps used to build a molecule is referred to as the synthetic route. When evaluating different routes, attention is often focused on the “key step(s)” or overall “strategy”, specifically, which bond-forming step(s) introduced the most structural complexity or represented the most innovation. If the routes under consideration have been experimentally tested, practical factors take precedence in the comparison. Common challenges include number of steps, yield, cost, safety and environmental sustainability of the process.^1^

Significant efforts to address these challenges are triggered by discoveries in both industry and academia. For example, producing inexpensive, high-quality graphene on a large scale from abundant source materials, using environmentally friendly processes, is essential for enabling the widespread and sustainable adoption of this so-called “wonder material”.^2^ In ths vein, several researchers have reported innovative findings related to high-yield graphene nanocomposites, emphasizing sustainable materials and green chemistry approaches for developing novel applications.^3–7^ In addition, graphene materials are considered instrumental in advancing the green revolution, therefore, many scholars have devoted substantial efforts on exploring graphene’s potential.^8^ Accordingly, the incorporation of graphene into materials such as cement, asphalt, and plastic composites has been observed and shown promise in improving durability.^9^ Graphene-based composites have also emerged as a new class of hybrid materials in which metal nanoparticles are uniformly distributed and prevented from agglomerating by being anchored onto graphene layers during the reduction process.^10^ Consequently, the future of graphene nanocomposites appeared promising, with growing interest in their fabrication and wide-ranging potential.^6^ In the context of environmental applications, graphene materials are commonly utilized as desiccants,^11^ thanks to their unique physicochemical properties. Their importance is mainly attributed to their biocompatibility.^12^ Accordingly, the pursuit of biodegradable materials and green chemical processes reflected a growing commitment to environmentally responsible innovation.^13^

Despite this milestone, many researchers now use derivatives of graphene, because of their surface functionalities and ease of dispersion in aqueous and organic solvents.^14^ One closest alternative to graphene, reduced graphene oxide (rGO) is gaining momentum.^15^ However, a significant drawback is emanating from lack of a cost-effective production of rGO^15^ and selecting synthetic approaches that are both economically viable and chemically diverse.^16^ Hence, to address these concerns current research has increasingly turned toward green synthesis methods.^17^

Green chemistry has emerged as a promising alternative for chemical synthesis^18^ and a step near the elimination of expensive toxic chemicals is the biologically chemical approach that offers significant environmental and economic benefits.^19^ Since the identifications of reductants derived from biological sources such as ascorbic acid^20^ and green tea^21^ as effective and environmentally benign alternatives to hazardous chemical reducing agents, in a drive for furthering the advantages of the approach, considerable effort has been invested in exploring more natural substances for the reduction of graphene oxide. Specifically, bacteria, fungi, yeast, or plants eliminate the need for hazardous chemicals, high temperatures, high pressure, or excessive energy input.^19^ Additionally, a wide variety of phytochemicals derived from natural sources—such as caffeine, spinach, grapes, carrot root, aloe vera, sugar cane juice, and protein isolate—have been studied for their potential in green reduction processes.^22^ Moreover, rice husk, a low-cost and abundant lignocellulosic biomass rich in cellulose, has been proposed as a promising sustainable precursor for graphene production.^9^ Beyond the biological sources described above, a few studies attempted to further suggest green applications of GO and rGO. One notable example is a recent study that explored the dispersion of GO within the biological structure of rose petals. The same work demonstrated that incorporating GO enhances the properties of the composite material and aligns with the core principles of green chemistry, promoting sustainability and reducing environmental impact.^12^ Another study highlighted the photocatalytic capabilities of rGO when hybridized with gold nanoparticles for the degradation of methyl orange dye under both UV and visible light.^10^

Scholars are confronted with two challenges. First, only a handful of studies focus on guarana and pomegranate in the development of rGO nanocomposite through a green, sustainable approach. Previous studies have demonstrated the successful reduction of GO to rGO using various plant extracts. Eucalyptus leaves, ginger, garlic, espand seeds, and green tea, for instance, have all served as natural reducing and capping agents, resulting in rGO with enhanced properties as confirmed through various characterization techniques.^23,24^ To date, only Botinelly Nogueira et al. have reported the green synthesis of silver nanoparticles (AgNPs) using guarana seed skin extract, as a source of both reducing and stabilizing agents.^25^ Additionally, a notable advancement was reported by Anagbonu et al., who were the first to develop a green, simple, and energy-efficient method for synthesizing few-layer graphene sheets from pomegranate peels.^26^ Additionally, the consensus among technical aspects and cost data is that although established methodologies exist,^27^ and these methodologies set the precedent to lower overall synthesis process it is unclear how universal these result were, as each synthetic route different economic weak points can be discerned which may ultimately increase the cost of the process. Indeed, there is a demand for developing low-cost synthesis methods, eliminating the arduousness of cost complications. Considering the above issue, and because of these confounding variables, we believe it is important to understand the different cost factors in detail, which is pivotal in a synthetic process, comparing the total synthesis processes economics for the same nanomaterials is nontrivial.

Aimed at overcoming the limitations of conventional inorganic synthesis methods, such as prolonged reaction times and multistep procedures, this study focuses on the development of rGO nanocomposite through a green, sustainable approach. A central aspect of this approach is the utilization of guarana as a green reducing agent, along with pomegranate biomass, to replace conventional chemical reagents with biodegradable alternatives, offering both ecological and economic advantages. Keeping this in view, the novelty of this study is 2-fold and (i) present a sustainable methodology for synthesizing rGO nanocomposites for use in separation processes, employing an innovative, environmentally friendly protocol, and (ii) inspired by the intrinsic advantages of the synthesis cost profile in this contribution, we further explore a large economic scope of same nanomaterials and studied their cost relationships. This comprehensive experimentally driven cost analysis gives vital information about how various factors affect the overall synthesis cost to best guide the choice of a specific rGO nanomaterial. To the best of our knowledge, this is the first study to incorporate the combined benefits of guarana and pomegranate biomass in rGO synthesis. It showcases how these readily available, plant-based materials can replace complex and resource-intensive laboratory methods while advancing environmentally responsible practices in inorganic synthesis. Moreover, the use of pomegranate biomass facilitates faster reduction reactions without compromising yield when compared to conventional methods. These promising preliminary results underscore the potential of pomegranate biomass to simplify synthetic protocols while prioritizing both ecological and economic sustainability in rGO production.

## 2 Experimental Section

The synthesis typically begins with a experimental designing phase, where suitable mechanisms and procedures are selected, followed by a trial-and-error phase.^28^ A synthesis pathway includes some essential information such as the steps involving precursors and target materials, their quantities, and the corresponding synthesis actions and attributes, arranged in the correct sequence. This represents the minimum information necessary to complete a synthesis route.^29^ A “step” can be defined either as a reaction step^30^ or a process step within a given synthetic route. Based on these definitions, we quantify the cost of a process step to assess the efficiency of various synthetic pathways (see details in the Supporting Information).

### 2.1 Selection of Reaction Times

Hydrothermal reduction of GO to rGO mostly occurs with heating times of 4 to 24 h, based on the extent of reduction needed, defect control, and application.^31–33^ Under hydrothermal conditions, GO self-assembles in an interconnected gel-like framework, thus structural control of porosity is very important in order to achieve high surface area and energy storage. The right deoxygenation levels and porous morphologies are attained by optimization of the temperature and hydrothermal reaction time. In researches that the latter was studied, it was found that the 4 h was the optimal time, where simultaneously the oxygen functional units were removed and the structural integrity was maintained.^34,35^ Higher reduction times led to more defects and damaged structure which is not ideal for a lot of applications. Thus, the current study explores 2 reduction times, at 4 and 16 h as a comparison.

### 2.2 Chemical Reaction Mechanism

The mechanism of reduction utilizing plant extracts to make rGO is nearly same in all cases.^36^ GO functional groups include −OH, −COOH and −O–. Secondary metabolites such as polyphenols, proteins, sugar compounds, alkaloids, and flavonoids play a vital role in the removal of oxygen functional groups from the GO surface. Polyphenols, which are abundant in plant extracts, react with a portion of the epoxide using the SN_2_ mechanism to open the oxirane ring. Similar nucleophilic attacks by polyphenols happen on hydroxyl and carbonyl groups, followed by water molecule removal.^37^ Hydrothermal reduction involves several processes.^38^ Hydrogen atoms, hydroxyl, epoxy, ether, and carboxyl groups terminate GO at its borders and basal plane. During hydrothermal reduction, GO undergoes ring-opening of epoxy groups to generate hydroxyl groups, followed by H^+^-catalyzed dehydration at moderate temperatures.

The synthesis mechanism of rGO from agricultural waste with ferrocene includes thermal decomposition and catalytic effects.^39^ The heating of biomass, which is made up of cellulose, lignin, and hemicellulose, evaporates water and volatiles, and causes partial decomposition of carbon compounds, enhancing porosity. Hemicellulose and cellulose experience extensive mass loss between 200–326 °C, releasing condensable (furan derivatives, anhydrosugars) and noncondensable (CO, CO_2_) volatiles. Mechanism of reduced graphene oxide growth. Carboxylic groups are removed thermally to reduce graphene during a short annealing step. During extended annealing, the removal of carboxylic and epoxy groups was accompanied by carbon loss and associated defect formation, thus resulting in the formation of CO_2_ and CO byproducts.^40^ At temperatures above 249 °C, ferrocene volatilizes, penetrating the porous carbon matrix, where it is reversibly oxidized to ferrocenium ions, which act as electron donors or Lewis acid catalysts. The ions catalyze secondary reactions, providing for electron transfer to residual carbon structures and oxidative rearrangement catalysis, while in situ acids that are formed by biomass pyrolysis reduce oxygenated groups via acid-catalyzed opening to diols followed by reduction to alkenes.^40^ The combination of ferrocene-mediated catalysis, thermal restructuring, and acid-supported deoxygenation facilitates the development of sp^2^ -hybridized graphene-like structures, which is confirmed by FTIR/XRD analysis.

### 2.3 Common and Alternative Synthetic Routes toward Synthesis of rGO

This work compares four different synthetic pathways. Figure 1 illustrates the schematic diagram of rGO synthesis processes.

**Figure 1:**
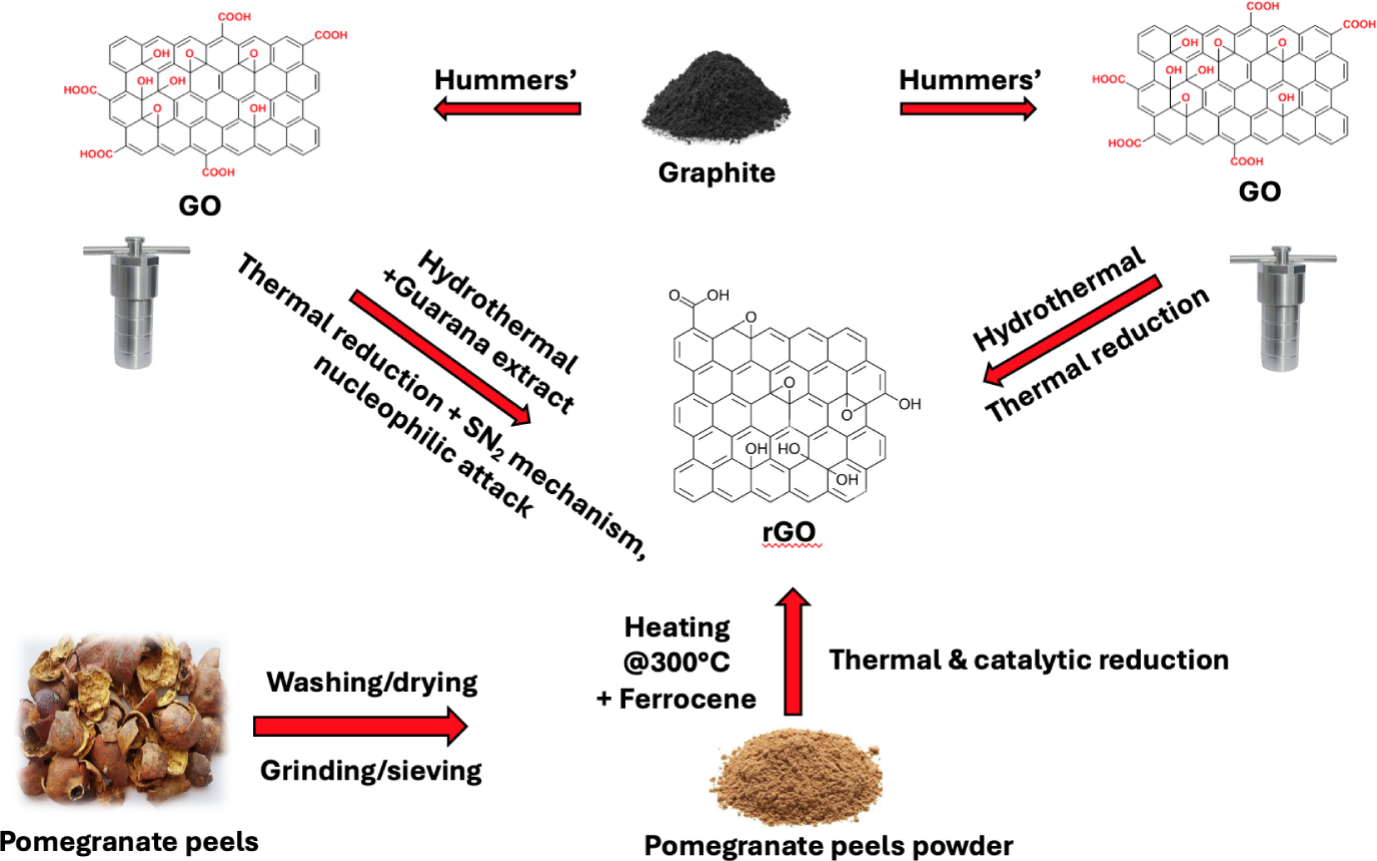
Schematic diagram of rGO synthesis processes.

To produce rGO, the first step is the synthesis of GO. Graphene oxide (GO) was synthesized by mixing 360 mL of sulfuric acid (H_2_SO_4_) with 40 mL of phosphoric acid (H_3_PO_4_) in a 9:1 ratio. Orthophosphoric acid helped inject the oxidation solution.^41^ Then, 3 g of graphite flakes were added, followed by the gradual addition of 18 g of potassium permanganate (KMnO_4_), raising the temperature to 40–50 °C. The mixture was heated to 50 °C in a water bath and stirred for 12 h, evolving into a paste. Afterward, 400 mL of distilled water was added, and stirring continued for another 30 min. Finally, 10 mL of 30 wt % hydrogen peroxide (H_2_O_2_) were added, causing a bright yellow color change and facilitating the reduction of manganese ions to soluble manganese sulfate and oxides. The resulting GO was washed with 200 mL of 37% HCl and distilled water until the pH reached about 6, and then freeze-dried for 48 h.

#### 2.3.1 First (Common) Synthesis

A specific amount of synthesized graphene oxide (GO) was ultrasonically dispersed in 20 mL of deionized water for 30 min. The reduction process was conducted in a hydrothermal autoclave at 180 °C for either 4 or 16 h. After the reaction, the resulting reduced graphene oxide (rGO) settled at the bottom of the autoclave, was centrifuged multiple times, and washed with deionized water to remove unwanted components. The final black powder was freeze-dried for 48 h.

#### 2.3.2 Second Synthesis—First Green Improvement

A specified amount of synthesized graphene oxide (GO) was ultrasonically dispersed in 20 mL of deionized water for 30 min. After sonication, 10 mL of guarana extract was added to accelerate reduction, and the mixture was stirred for extra 30 min for uniformity. The reduction process took place in a hydrothermal autoclave at 180 °C for 4 h. The resulting reduced graphene oxide (rGO) settled at the bottom, was centrifuged multiple times, and washed with deionized water to remove impurities. Finally, the black powder was freeze-dried for 48 h.

#### 2.3.3 Third Synthesis—Second Green Improvement

Pomegranate peels were dried in an oven for 2 h and then ground into a fine powder. The rGO was synthesized by oxidizing the peels (0.5 g) with ferrocene (0.1 g) at 300 °C under muffled atmospheric conditions. The resulting black product was thoroughly washed with deionized (DI) water to remove any unreacted Ferrocene.

### 2.4 Characterization

Fourier Transform Infrared (FTIR) analysis was employed to characterize the functional groups of the materials. The laboratory utilized a PerkinElmer Frontier attenuated total reflectance Fourier transform infrared spectrometer (ATR-FTIR, ZnSe) for the analysis, operating at a nominal resolution of 2 cm^–1^ and covering a spectral range of 4000–550 cm^–1^. X-ray diffraction (XRD) was conducted to assess crystallinity over a 2θ range of 5–45° using a BRUKER D8 FOCUS X-ray diffractometer, equipped with CuKα radiation (λ = 0.154 nm). The materials’ morphology was analyzed using a JEOL JSM6390LV scanning electron microscope (SEM). Small Angle X-ray Scattering (SAXS) was used as a structural characterization technique to analyze the architecture of the composites. SAXS experiment was performed in a SAXS rig system provided by JJ X-ray Systems with a CuKa X-ray source (λ = 0.15189 nm). The apparatus consists of a Pilatus *R*-300 K detector, vacuum chamber, pinholes to collimate the beam, and an X-ray source. All measurements were corrected for background radiation and transmission. The sample–detector distance was set at 1.5 m and each sample was measured for 3600 s.

## 3 Results and Discussion

In the context of sustainable development, this manuscript presents an innovative approach to rGO synthesis by utilizing guarana and pomegranate biomass, highlighting the method’s inherent simplicity and environmental compatibility. This green strategy capitalizes on the advantages of eco-friendly rGO in water-based solvent systems, marking a significant step forward in sustainable synthesis practices. The newly developed protocol ensures low cost, high yield, and minimal environmental impact, clearly demonstrating the practicality and versatility of using accessible materials like pomegranate to both streamline the synthesis process and enhance reaction efficiency.

### 3.1 Characterizations

The morphology, composition and functional groups were measured and identified by XRD, SEM and FTIR analysis.

#### 3.1.1 Fourier Transform Infrared Analysis

The O–H stretching vibrations in the 3500–3200 cm^–1^ (Figure 2) range belong to the carboxyl and hydroxyl units. These hydrophilic functional moieties bearing oxygen offer GO samples with high dispersion in aqueous elements.^42^ The peak around 1710 cm^–1^ corresponds to the ketone group (C=O), whereas the 1520 cm^–1^ peak represents the C=C.^43^ Lastly, the band at ∼1100 cm^–1^ represents the C–O stretching of the epoxy groups. These data indicate that graphite was effectively oxidized.

**Figure 2:**
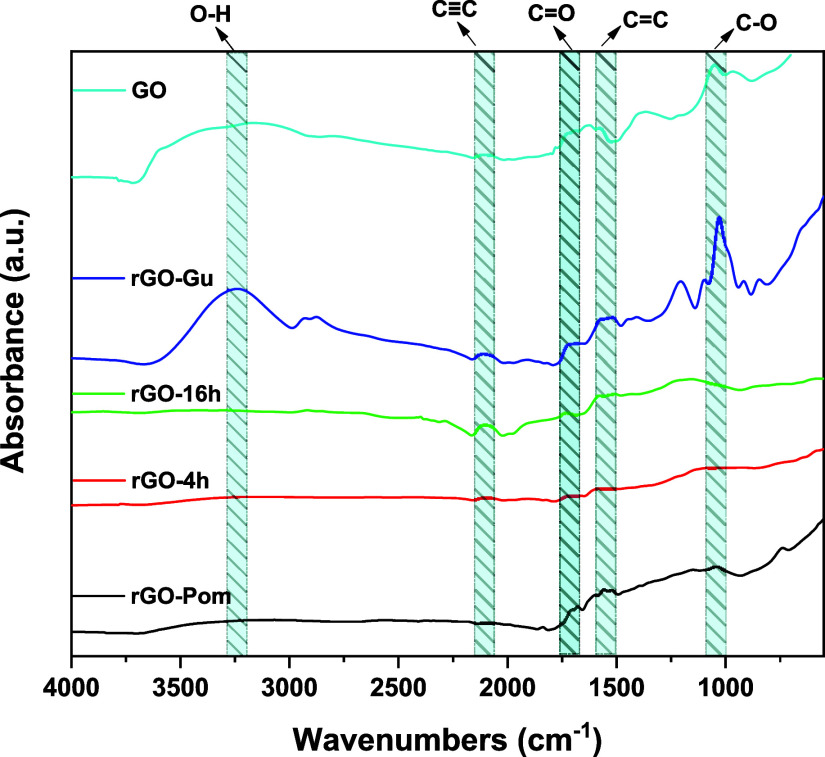
FTIR spectra of GO, rGO (4 h), rGO (16 h), rGO-Gu, and rGO-biomass.

For rGO samples, the intensity of the majority of the oxygenated groups’ peaks decreases. The band observed at 1100 cm^–1^, corresponding to the carboxyl groups (C–O), indicates the occurrence of decarboxylation and the removal of −OH units.^44^ This removal leads to the formation of conjugated sp^2^/sp^3^ bonds in the rGO samples, effectively restoring aromaticity by reestablishing sp^2^ bonds within the graphitic structure.^45^ Also, a new peak is introduced at ∼2100 cm^–1^. which can be attributed to the presence of alkyne groups (C≡).^46^ GO-Gu seems to be reduced the least, since the peaks of its oxygen-containing functional groups appear still to be sharp and with higher intensity, when compared to the other produced rGO. The latter comes in agreement with XRD analysis.

#### 3.1.2 X-ray Diffraction Analysis (XRD) and Small Angle X-ray Scattering (SAXS)

A single peak was detected in GO (Figure 3a) at 10.34° (001) with a *d*-spacing of 0.86 nm, calculated using Bragg’s law, indicating the intercalation of water and the formation of carboxylic groups within the graphite sheets during oxidation. After the reduction process, the reflection peak at 10.9° disappeared, leaving a broad peak centered at 23.45° (002), 23.87° (002), and 21.62° (002), corresponding to *d*-spacings of 0.38, 0.37, and 0.41 nm for rGO synthesized via hydrothermal treatment for 4 h, 16 h, and with Guarana extract, respectively. Additionally, the *d*-spacing of rGO significantly decreased after reduction, suggesting the removal of oxygen-containing functional groups.^47^ For the production of rGO through the catalytic oxidation of pomegranate peels, a more amorphous structure and a peak at 23.27° can be observed, showing a *d*-spacing of 0.38 nm. The lower interlayer distance between graphene layers suggests a larger degree of reduction and a *d*-spacing closer to graphite (0.33 nm, at ∼26.5°). Thus, rGO-Gu underwent the least reduction of its oxygen-type functional groups, while rGO-Pom along with rGO materials obtained from hydrothermal method, are closer to graphite structure interlayer distance.

**Figure 3:**
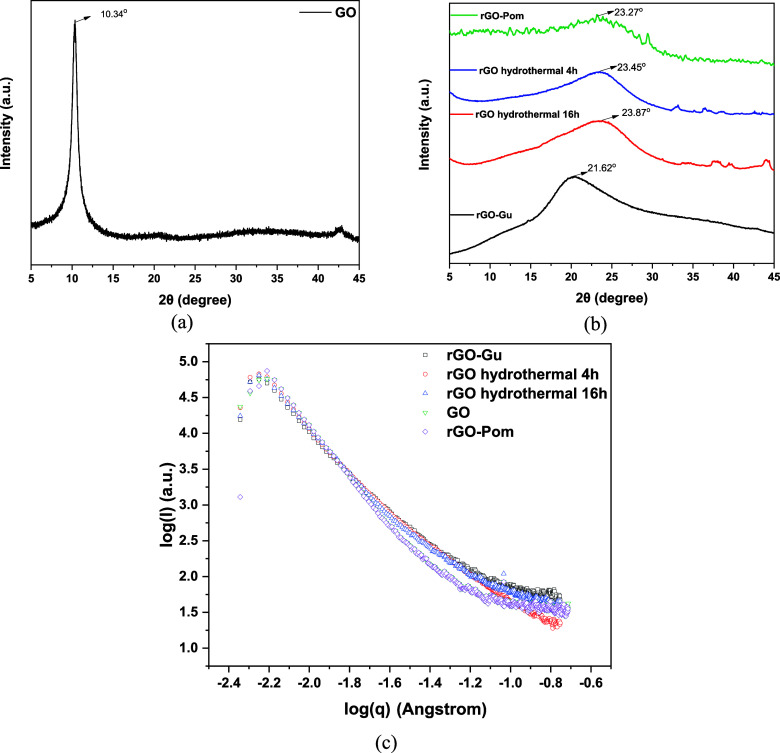
(a) XRD spectrum of GO, (b) XRD spectra of rGO derivatives, and (c) SAXS profiles of GO and rGO derivatives.

According to the calculations of Guinier, for aggregate systems consisting of small polydisperse particles, the scattering curve can be computed by the following equations (eqs 1–3):

1 $$I_{q}=\frac{A}{{\left(1+q^{2}r^{2}\right)}^{2}}$$

where *q* indicates the scattering vector, *r* is a measure of the particle radius, and *I*_*q*_ denotes the scattering intensity.

2 $$I_{q}=I_{0}\exp\left(-\frac{{R_{g}}^{2}q^{2}}{3}\right)$$

where *R*_*g*_ is the radius of gyration and *I*_0_ is the intensity at zero scattering angle (*q* = 0).

*D*_*f*_ is calculated from the slope of the straight section in the log­(*I*) versus log­(*q*) plot of the SAXS pattern

3 $$I\left(q\right)\propto q^{-D_{f}}$$

As it can be shown in Table 1, the comparison of radius of gyration (*R*_*g*_) and fractal dimension (*D_f_*) values, alongside the SAXS intensity profiles (Figure 3b), suggest that rGO-Biomass exhibits a nanostructure similar to the other reduced graphene oxide (rGO) materials.

**Table 1:** Comparison of Radius of Gyration (*R*_*g*_) and Fractal Dimension (*D*_*f*_) Values

	*R*_*g*_ (nm)	Fractal dimension (*D*_*f*_)
rGO-GU	17.7	1.8
rGO-4 h	20.3	2.1
rGO-16 h	18.4	1.9
GO	15.6	1.8
rGO-biomass	15.7	1.8

SAXS was used as a structural characterization technique to analyze the architecture of the composites. To begin with, rGO-Pom has an *R*_*g*_ of 15.7 nm and a *D*_*f*_ of 1.8, which are nearly identical to GO (15.6 nm, *D*_*f*_ = 1.8) and comparable to rGO-Guarana (17.7 nm, *D*_*f*_ = 1.8) and rGO-16 h (18.4 nm, *D*_*f*_ = 1.9). Despite modest changes in *R*_*g*_, the constant *D*_*f*_ values show that the materials have comparable fractal surface patterns and aggregate forms. Moreover, the SAXS patterns of rGO-Pom align with the intensity decay of the other rGO samples. Especially in the intermediate and high-q areas, where it is showing that they all have similar nanoscale properties such as surface roughness and internal structure. This proximity shows that rGO-Pom’s synthesis route yields a nanostructure comparable to that of other rGO materials.

#### 3.1.3 Scanning Electron Microscopy (SEM)

Morphological characteristics of GO and rGO samples were investigated by SEM examination, as illustrated in Figure 4. rGO-Gu is wrinkled (Figure 4a), and layered structure. Its framework has a smooth surface with little folding, which is attributable to the presence of new organic functional groups on the surface following effective oxygen group elimination by guarana extracts, leading to the partial restoration of the graphene structure.

**Figure 4:**
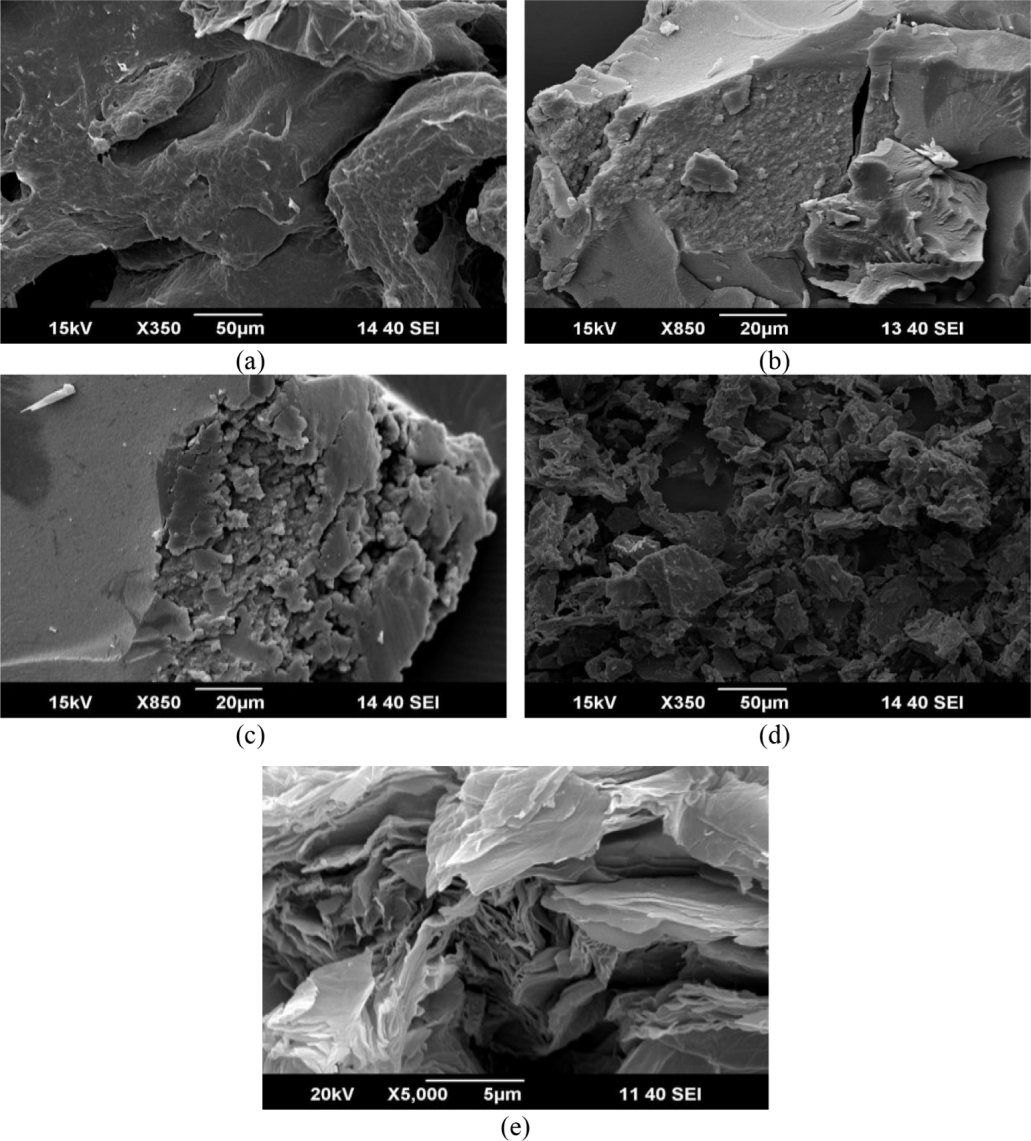
SEM images of rGOs from different synthetic routes: (a) rGO-Gu, (b) rGO (16 h), (c) rGO (4 h), (d) rGO-Biomass, and (e) GO.

For both rGO obtained from hydrothermal process (Figure 4b,c), they seem irregularly shaped, with stacked graphene layers. The rough texture and folded edges provide evidence of the reduction process, indicating a decrease in the oxygen content and partial restoration of the conjugated graphene structure. Lastly, rGO-Biomass (Figure 4d) shows a similar morphology to rGO-Gu, with a smooth surface area and little folding. Instead, Figure 4e shows a picture of GO with clearly established wrinkled sheets layered on top of one another.

### 3.2 Optimization of Synthetic Route

To reduce costs and optimize the synthetic route for rGO, we first aimed to enhance the common synthesis method. We initially employed synthetic path A (16 h, 8 steps), which is similar to synthetic path B (4 h, 8 steps). Even after changing the duration from 16 to 4 h, the synthesis cost remained high. To further explore the impact of material costs on the synthesis process, we implemented synthetic path C (using guarana, 9 steps). This adjustment resulted in a significant improvement and effectively reduced costs. However, it is important to note that the material costs do not increase linearly with the number of synthetic steps. Figure 5 indicates that the cost of materials of the reduction process is lower as opposed to that of the preparation process. This disparity is more pronounced for rGO produced through common synthetic pathways (4 and 16 h) and the optimized synthetic route using guarana. Specifically, the material cost for the reduction process is reduced by 91% for the common 16-h and guarana methods, and by 90% for the common 4-h method, in comparison to their respective preparation processes.

**Figure 5:**
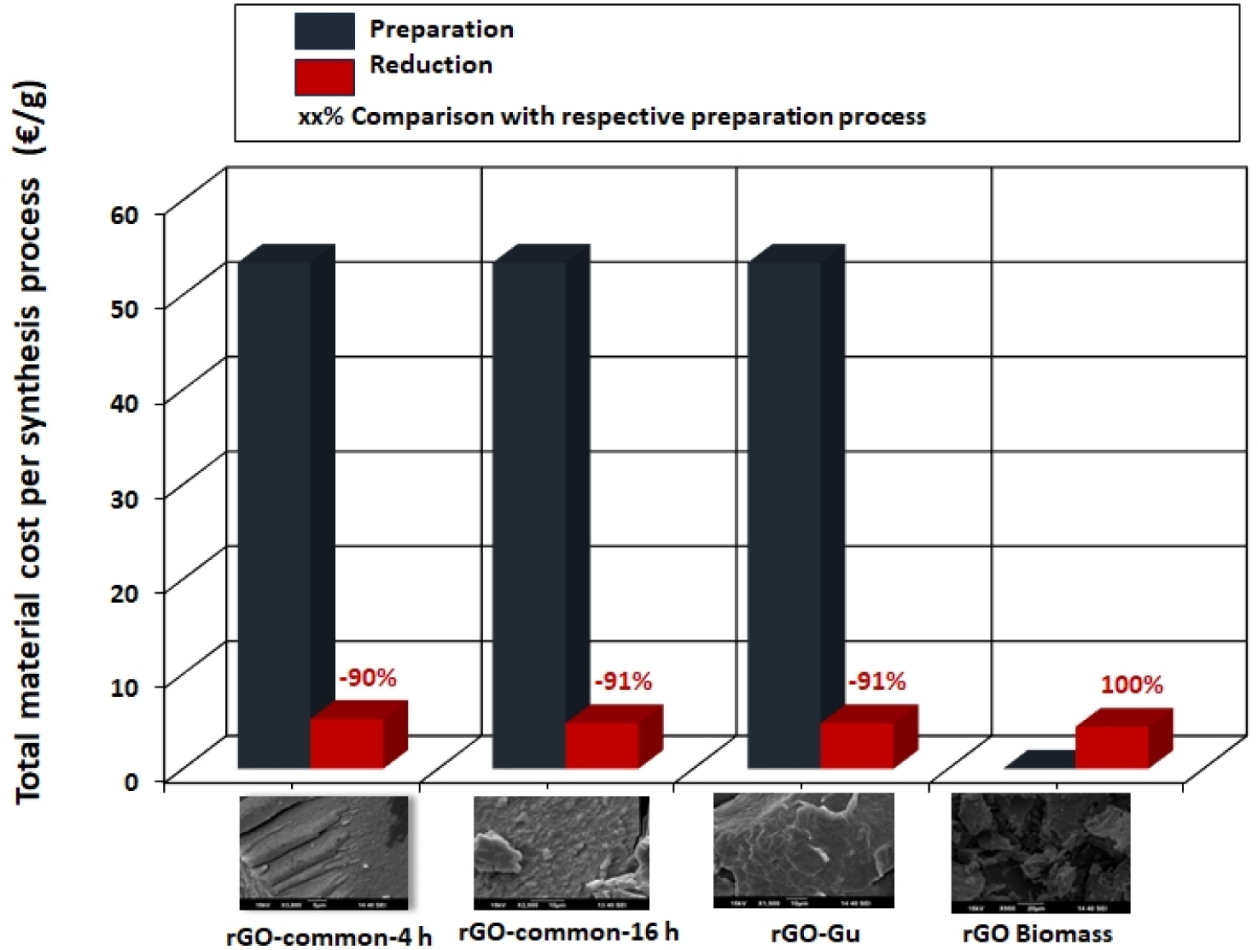
Material cost per rGO material for preparation and reduction processes.

This significant difference is largely attributed to the high material cost of GO during preparation. By employing the faster method (synthetic route D, rGO biomass), material costs can be decreased by nearly 100% for the preparation process. For synthetic routes A, B, and C, the production of GO as a preparation step is both energy- and labor-intensive (see Table 2 and Figure 6a,b). Considering labor hours and the corresponding hourly labor costs, we calculated the total labor costs associated with synthesizing the four rGO materials (Figure 6a). The findings reveal that labor costs for the preparation process are significantly higher -up to 96%- as it requires several manual labor hours compared to the reduction process. In the case of rGO biomass (synthetic route D, which consists of three steps), the labor savings amount to nearly 100% for both processes. This is primarily due to the reduced number of labor hours required during both the preparation (drying) and reduction processes, which is expected given that synthetic route D is a faster process.

**Table 2:** Cost Profiles of rGO Materials

Synthesis process	Material cost (€)	Labor cost (€)	Energy cost (€)	Energy cost (Wh)	Maintenance cost (€)	Depreciation cost (€)	Total after tax and discount (€)	Cost/g (before tax and discount) (€)
GO (preparation)	53.41	24.61	6.74	42.125	0.40	1.92	10.74	15.89
rGO 4 h (reduction)	4.80	1.41	6.58	41.125	1.48	13.04	34.27	170.15
rGO 4 h total	58.21	26.02	13.32	83.250	1.88	14.97	139.01	186.04
rGO 16 h (reduction)	4.80	1.41	9.65	60.313	1.68	13.04	38.20	232.75
rGO 16 h total	58.21	26.02	16.39	102.438	2.08	14.97	142.94	248.64
rGO Guarana 4 h (reduction)	5.24	1.41	6.58	41.125	1.48	13.04	34.81	30.91
rGO Guarana total	58.65	26.02	13.32	83.250	1.88	14.97	139.54	46.80
rGO Biomass (reduction)	4.44	0.08	0.01	0.063	0.00	0.16	5.64	15.45
rGO Biomass (preparation)			0.06	0.375	0.00	1.16	1.47	4.03
rGO Biomass total	4.44	0.08	0.07	0.438	0.01	1.31	7.11	19.48

**Figure 6:**
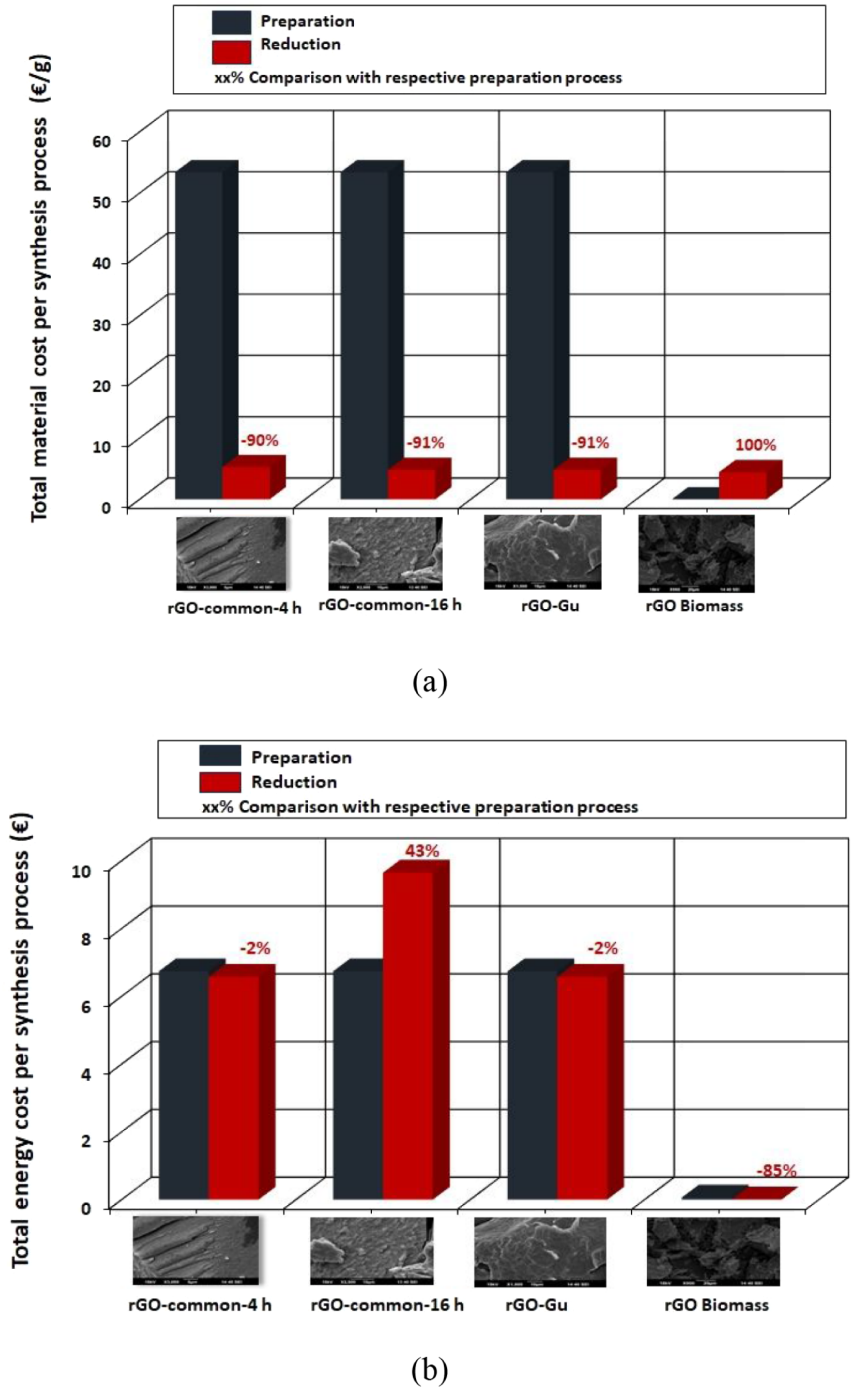
(a) Labor cost per rGO material for preparation and reduction processes, and (b) energy cost per rGO material for preparation and reduction processes.

Figure 6b illustrates the calculated energy costs associated with the synthesis of the four rGO materials through both preparation and reduction processes. It is evident that the common rGO 16-h reduction process involves energy-intensive activities, contributing to a higher total energy cost. This significant difference is primarily due to the substantial energy consumption of the preparation equipment. The disparity is particularly pronounced in the case of rGO biomass, where the energy cost for the reduction process is 85% lower compared to the preparation process.

### 3.3 Selecting Cost-Optimal Pathways

Figure 7 presents a comparison of the reduction costs (€/g) against the number of synthesis steps and Figure 8 illustrates total process duration per rGO material for preparation and reduction processes.

**Figure 7:**
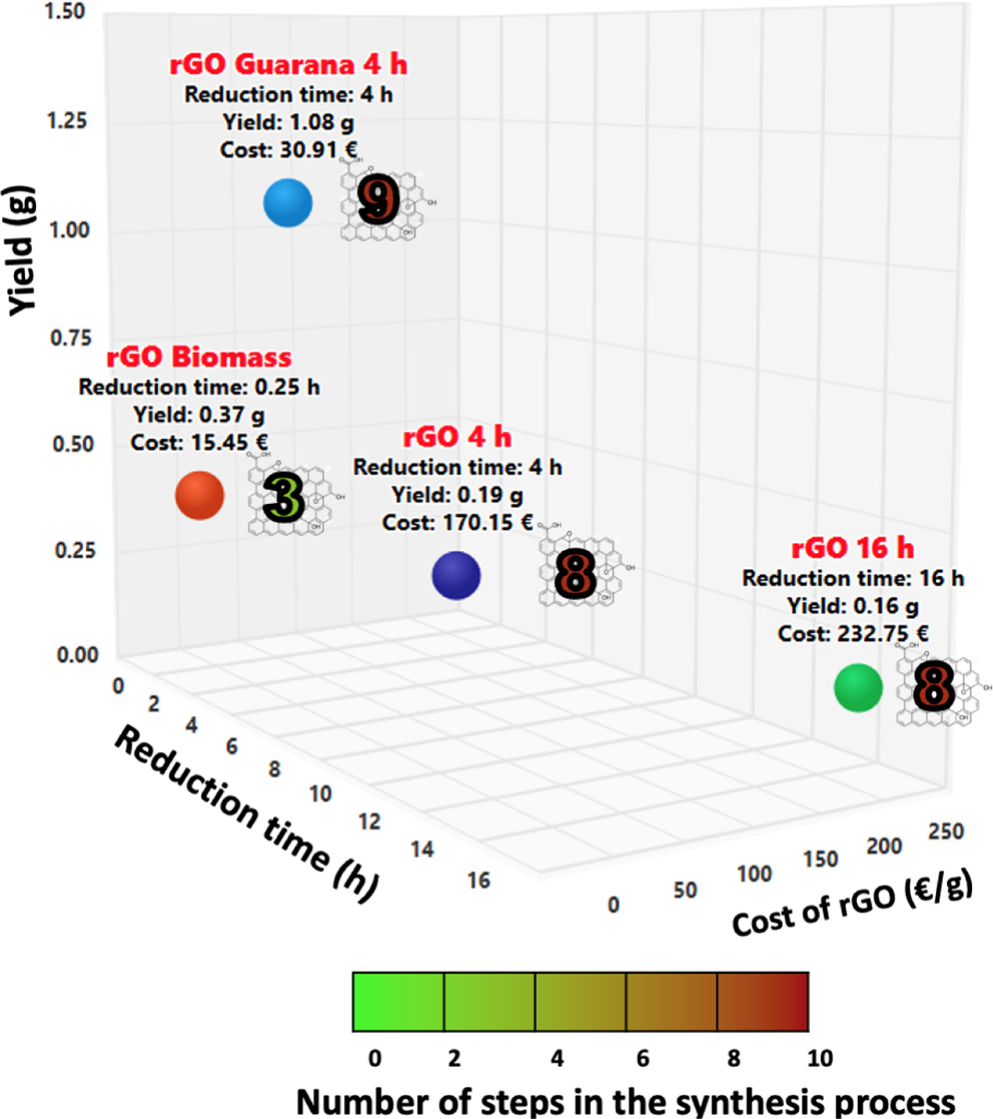
Evaluation results of rGO synthetic routes (the horizontal axes represent the reduction time needed for each synthesis and the cost of the synthetic route; the vertical axis indicates the overall yield of each route; each point is color-coded according to the number of synthetic steps and labeled with its corresponding route number).

**Figure 8:**
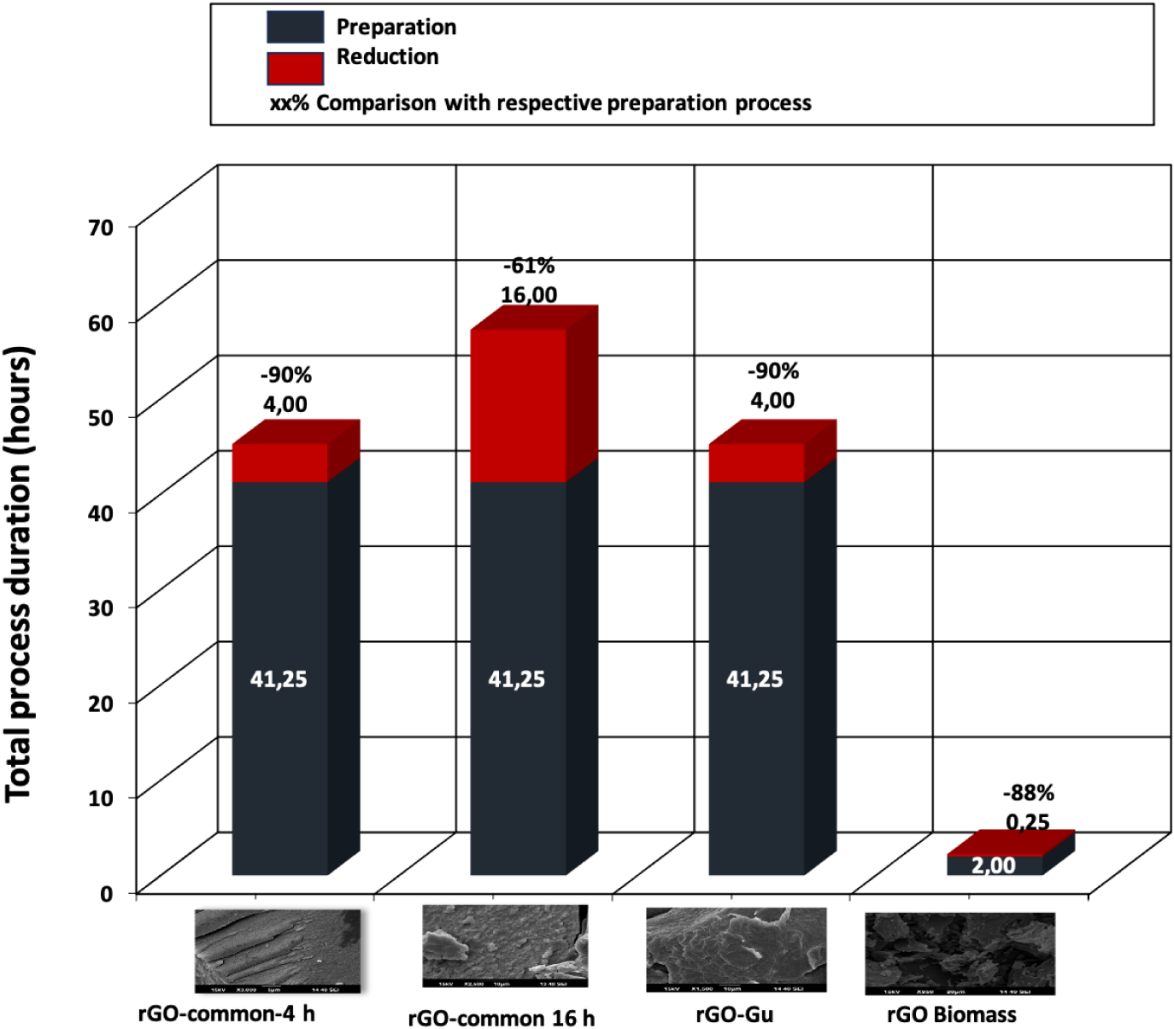
Total process duration per rGO material for preparation and reduction processes.

Figure 8 shows that the cost of materials (CM, cost/g) does not increase linearly with the number of synthetic steps. Route B, which is nearly identical to Route A, requires less time (4 h). However, both Routes A and B involve 8 steps, resulting in total costs of 232.75 € for Route A and 170.91 € for Route B. Literature suggests that rGO typically requires more than eight steps (e.g., Amir Faiz et al.^48^ identified 10 steps, while Tadjenant et al. reported 9 steps.^49^ In this study, by optimizing the synthetic procedures, Route D demonstrates the lowest cost among the pathways, with only 3 synthesis steps yielding a total of 0.365 g. In contrast to the other routes, Route D is conducted as a single multistep synthesis, designed to minimize the labor and energy needed for the synthesis processes.

Additionally, Route D offers several advantages: (i) the product (rGO) is obtained without the need for GO, simplifying the preparation of additional materials; (ii) it employs a straightforward pathway that facilitates rGO preparation; and (iii) it avoids harsh conditions. As a result, compared to the previously reported synthesis processes, which often involve lengthy reaction times and complex steps, the synthesis of rGO via this route is both more efficient and convenient, leading to lower costs. This laboratory-scale synthesis has proven to be cost-effective, and its quantitative data can be leveraged to highlight the benefits of other simplified pathways for low-cost and environmentally friendly synthesis. Overall, the findings underscore the significance of optimizing synthetic procedures to enhance yield and reduce the number of individual synthetic steps.

### 3.4 The Risk/Return Efficient Frontier

Each of the studied synthesis routes presents different level of risk. Figure 9 illustrates the curves representing various rGO synthetic pathways, based on the synthesis methods employed and their associated economic returns. The complexity of the synthetic pathways serves as the key variable, influencing costs and outcomes. The vertical axis depicts the expected economic returns, while the horizontal axis represents the number of steps in the synthetic pathways. Each curve reflects the economic returns achievable under specific conditions.

**Figure 9:**
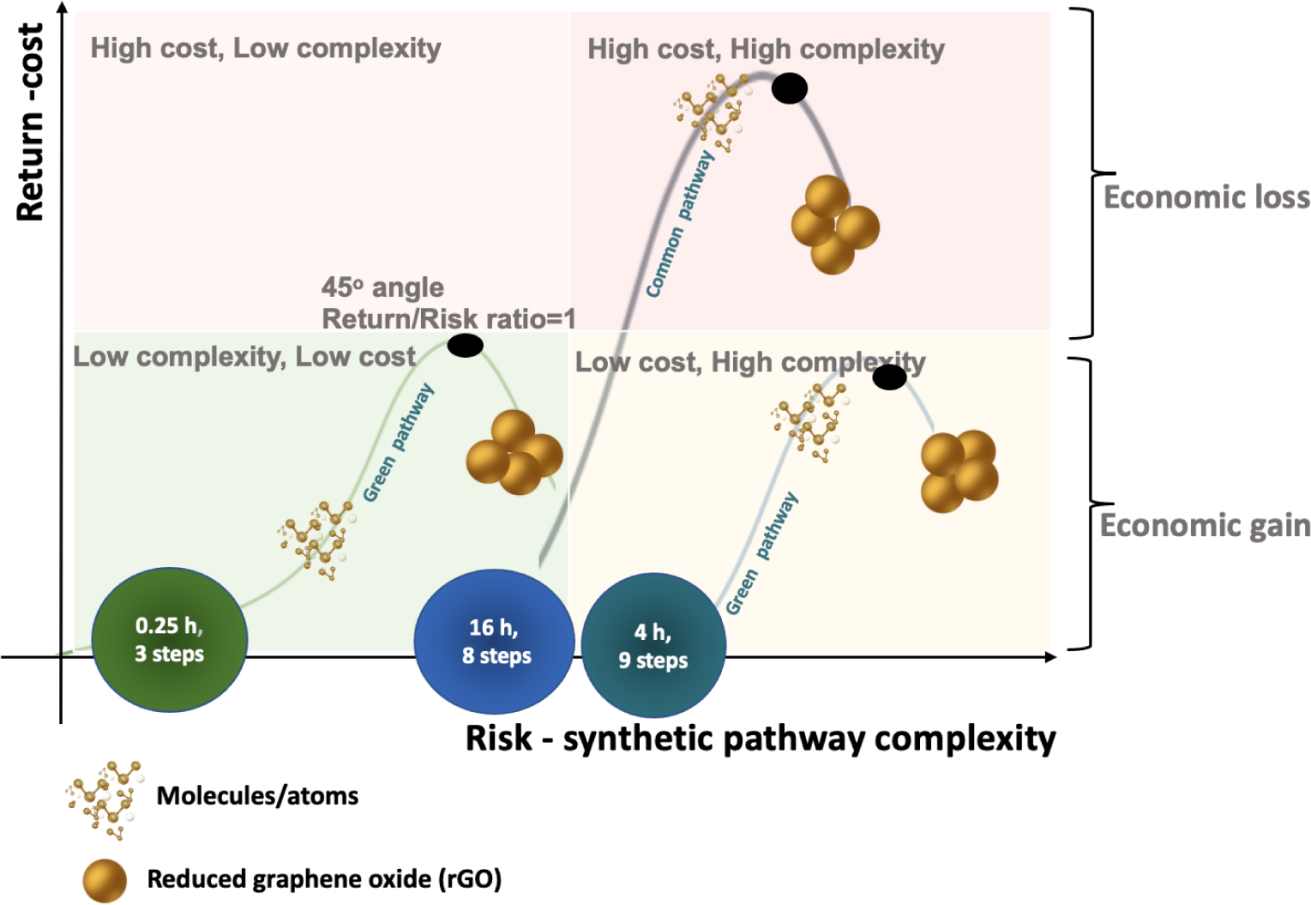
Risk (synthetic pathways)–return (cost) profiles for rGO.

The quadrants present a risk-return combination: Upper left quadrant: [Low complexity/High cost), Lower left quadrant: [Low cost/Low complexity], Upper right quadrant: [High cost/High complexity], Lower right quadrant: [High complexity/Low cost]. The four quadrants correspond to those of the complexity- economic returns matrix, with the lower left one (Low/Low complexity) being the target for optimum results. These quadrants align with the complexity-economic returns matrix, where the lower left quadrant (Low complexity/Low cost) represents the optimal outcome.

Each of the examined synthetic pathways presents a distinct level of risk. Figure 9 illustrates the risk-return curves for four synthesis routes, based on the number of process steps they involve (risk) and the cost-efficiency they offer (return), thereby covering a range of possible combinations. The variable of interest is the number of steps (X), which influences both the probability (p) and the cost reduction factor (crGO) shown in Figure 9. On the graph, the vertical axis represents the expected return in terms of synthesis cost (*c*_expect_), while the horizontal axis indicates the associated risk. Each curve reflects the potential returns achievable for a given risk level. The pathways rGO guarana/rGO 4 h and rGO 16 h are associated with less favorable outcomes. Specifically, rGO guarana/rGO 4 h lies in the upper-right quadrant (high cost – high complexity), while rGO 16 h is positioned in the lower-left quadrant (low cost – low complexity), indicating limited returns in both cases. Notably, all curves begin to flatten beyond a certain point, approaching a slope parallel to the *x*-axis. This behavior suggests that additional increases in risk yield only marginal gains in return. The black squares on each curve denote the point at which the slope is 45°, representing a return-to-risk ratio of 1. Prior to this point, returns increase faster than risk (ratio >1); beyond it, the efficiency diminishes (ratio <1). Thus, optimal returns are achieved just before reaching this critical point. According to Figure 9, the rGO Biomass pathway yields the most favorable performance, offering the highest economic return with the least synthetic complexity.

Table 2 outlines the cost profiles for the different rGO synthesis processes analyzed. To determine the cost profiles of each rGO material, the cost savings for each method were assessed, taking into account raw material costs, energy costs, labor costs, maintenance costs, and depreciation costs.

The cost of raw materials ranges from 4.40€ to 58.65€ and serves as the primary cost driver for the TCO in the synthesis processes of rGO Guarana, as well as common rGO synthesized in 4 and 16 h. Figure 10 illustrates the total synthesis costs for the four synthesized materials. It is evident that the common rGO synthesized over 16 h is the most expensive when compared to the other rGO materials. The material cost contributes the most to the total synthesis cost, followed by labor, depreciation, and energy costs, respectively. Additionally, Figure 10 shows that the TCO of the process is significantly influenced by the reduction process, with rGO Guarana and rGO biomass emerging as the most favorable options.

**Figure 10:**
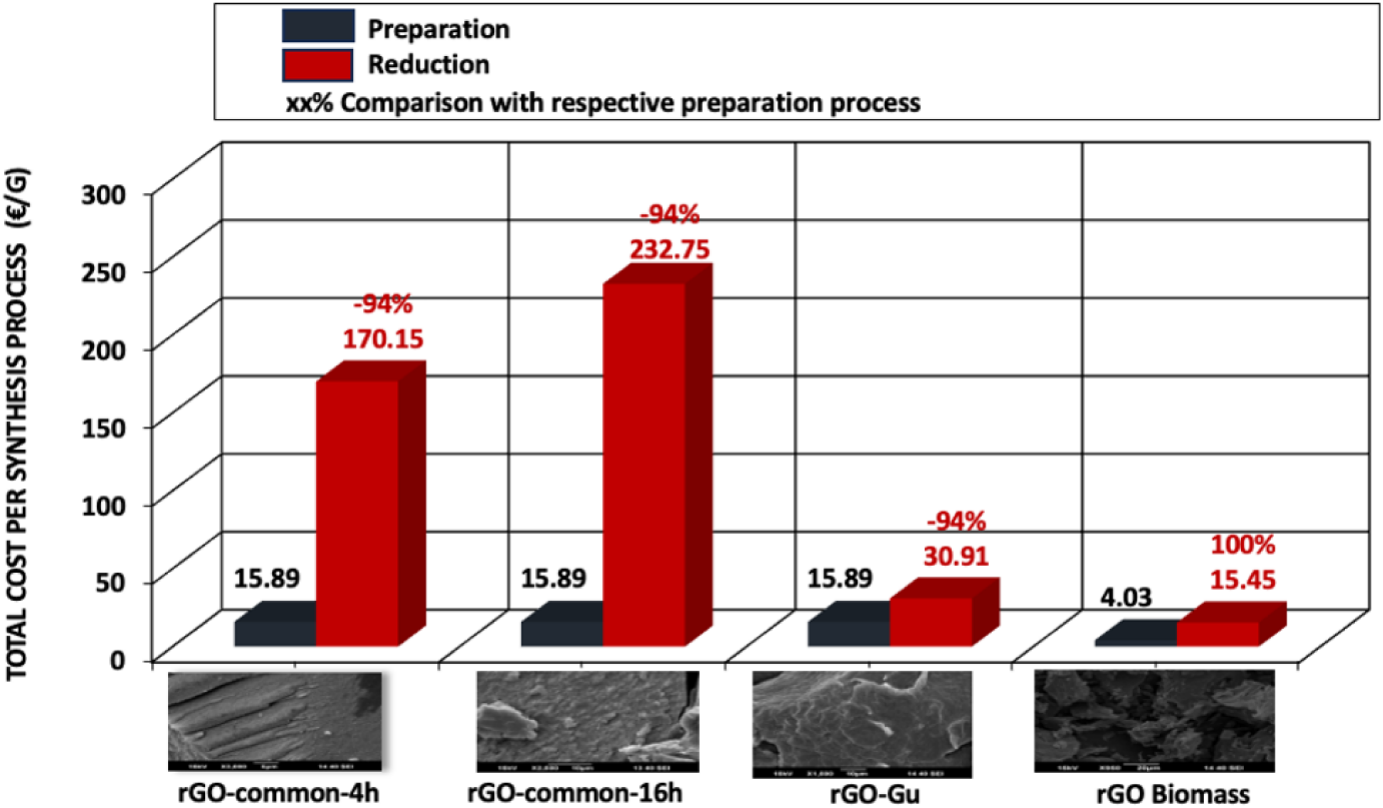
Total synthesis cost per rGO material for preparation and reduction process.

Figure 11 displays the synthetic pathways through which 1 g of the final product (represented by the yellow nodes on the right) is produced from various synthesis methods.

**Figure 11:**
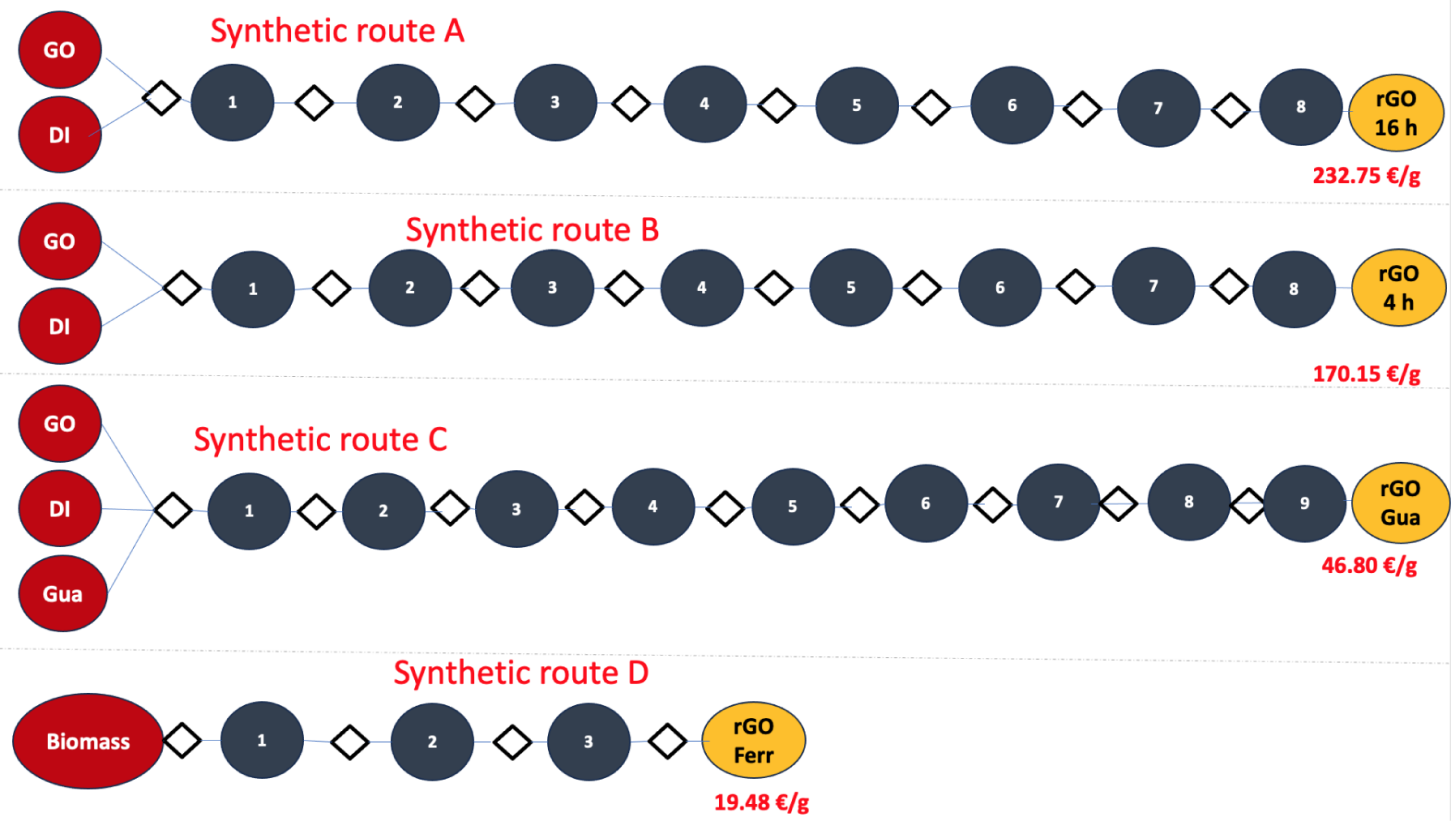
Effects of path structure and cost parameters on the cost of synthesis.

### 3.5 Key Strategies of Innovations

This study identified the key variables impacting the synthesis costs of rGO routes at the laboratory scale. Additionally, it employs a scalable approach capable of handling multiple data points and addressing the complexity of the issue. The most significant findings are (i) a new model was developed to assess the total cost of ownership (TCO) for rGO, identifying the cost parameters that constitute the TCO, (ii) none of the found cost parameters are unique to rGO, allowing the model to be generalized for other materials, (iii) this is the first instance where synthesis costs have been included in the evaluation of rGO.

The TCO model was used to quantitatively assess the actual requirements and economic potential of different synthesis processes. Despite its advantages, the study highlighted a lack of information regarding fundamental cost factors in the synthesis process. While this finding may be specific to the examined case studies, it emphasizes the need to consider synthetic steps during the design phase rather than solely concentrating on the synthesis process itself. Reducing the number of synthetic steps can significantly lower process costs. Solvent designs driven by economic factors often lead to reduced environmental impacts due to a more streamlined synthesis pathway. An efficient process not only improves economic outcomes but also benefits the environment; lower costs typically indicate reduced energy consumption, less waste, and reduced material requirements.^50^ Ultimately, redesigning the synthetic route (route D) led to a marked increase in synthesis efficiency, achieving an overall yield of 0.365 g with a three-step linear sequence, compared to the initial method. Notably, all transformations in this synthesis can be performed using neat, green processes.

Quantitative assessments revealed that the cost of the newly proposed route (19.48 €/g) is 18 times lower than the common 16-h route (232.75 €/g), demonstrating a substantial improvement in sustainability achieved through this work. Creating an effective cost profile relies on access to comprehensive information. This research has created and applied a TCO model that goes beyond basic cost comparisons to offer a comprehensive cost overview of the synthesis process. While labor and energy costs are essential components of the synthesis process, they were minimal for synthetic route D (rGO biomass). Therefore, a deeper investigation into these factors and the variations between the two processes may indicate that the primary parameter influencing TCO is associated with the process and the selected synthetic route.

Implementing a green chemistry approach can be a complex process due to the primarily qualitative nature of its principles. This indicates that there is no universal metric or minimum criteria for labeling a process as “green.” Green chemistry starts with researchers choosing to explore more sustainable alternatives in their process design.^51^ However, a sustainable chemical process depends on both environmental and economic (or societal) factors and benefits. While economic design can lead to significant advancements, it may be limited if the process involves highly toxic or persistent chemicals. As a result, the balance between economic and environmental factors often tends to prioritize economic considerations.^50^

As a result, these “hidden costs” need to be carefully balanced against the benefits from other areas to make an informed decision about which synthetic route genuinely results in the most sustainable process.

### 3.6 Outlook and Prospects for Future Scopes

Recommendation #1: Incorporate cost factors into the synthesis evaluation equation. Financial criteria should be considered when managing nanomaterial technology, as cost is a crucial element in decision-making regarding nanomaterial strategies. Paying adequate attention to these factors can enhance assessment processes, facilitating collaboration among stakeholders and fostering clearer discussions about technological innovation.

Recommendation #2: Standardize labeling for nanomaterial synthesis processes. Although new synthesis processes are frequently published, most lack information about produced quantities, synthesis costs, and the environmental sustainability of the processes. Establishing a predefined structured format for publishing this information will help create a global database that consolidates knowledge on nanomaterial synthesis.

Recommendation #3: Implement the ABC method for evidence-based management. Utilizing the ABC model is advisable due to its capability to reveal indirect costs and aid lab managers in making informed decisions. The previous communication gap between research scientists and economists has led to inconsistencies in prior reports; therefore, more emphasis should be placed on funding projects that encourage dialogue between these two groups on a global scale.

The complexity of the synthesis process often limits its potential for substantial economic returns. The primary goal is to maximize profits while minimizing risk, which involves reducing the use of toxic materials and streamlining the number of steps in the process. This strategy serves as a foundation for developing synthetic pathways that balance reduced risk (fewer chemicals and simplified routes) with enhanced benefits, both economic and environmental. By linking risk management with positive outcomes, this approach aims to support informed decision-making. Over time, optimizing synthetic pathways can become a pivotal strategy, reflecting long-term environmental and economic advantages while addressing the challenges associated with conventional, riskier methods.

## 4 Materials and Methods

Two common synthetic routes, known for their multistep complexity, were optimized by introducing guarana as a greener reducing agent. The goal is to develop a practical, robust, environmentally responsible synthesis, with particular attention to cost-effectiveness. By reducing the number of process steps from eight to three through key modifications, this study proposes a scalable, safe, fast, and green method for synthesizing rGO, achieving both time and cost savings. The selection of these four rGO synthesis routes serves three main purposes: (i) to compare the cost profiles of multistep synthesis processes, (ii) to identify the most efficient and green synthetic pathway, and (iii) to evaluate these processes in greater detail to provide more reliable cost information for each pathway.

### 4.1 Framework

This research aims to evaluate low-cost and green approaches at the lab scale. The synthesis process is divided into two main categories: (i) planning and (ii) design. It is further organized by tasks and methods, outlining step-by-step activities for achieving the research objectives. According to Gkika et al.,^52^ the complexities of managing the synthesis process can only be fully understood through Activity-Based Costing (ABC) and other detailed assessments. The ABC method gathers information about all activities (steps) involved in synthetic routes to evaluate cost-effective decisions for producing reduced graphene oxide (rGO). Furthermore, the application of green chemistry principles (GCPs) can further enhance economic performance. GCPs can be grouped into three main areas: Input selection and reduction encompasses 1 (waste prevention), 2 (atom economy), 7 (use of renewable feedstocks), 8 (reduction of derivatives), and 9 (catalysis). Sustainable design involves 4 (designing safer chemicals), 6 (energy efficiency), and 10 (design for degradation).Safety management includes 3 (less hazardous chemical synthesis), 5 (safer solvents and auxiliaries), 11 (real-time pollution prevention analysis), and 12 (inherently safer chemistry for accident prevention).^53^

From an economic and business perspective, the sustainable development goals (SDGs) advocate for chemical reactions that achieve higher yields while using smaller amounts of feedstock to produce the same quantity of product, aligning with SDGs 9 and 12. Additionally, reducing the number of synthetic steps often leads to faster product manufacturing, increased plant capacity, and savings in both energy and water, which are also relevant to SDGs 9 and 12.^53^ The application of green chemistry principles across four synthetic pathways is outlined in Table 3. Principle #3: Safer Synthetic Processes: This principle emphasizes that design must account for all potential factors that could negatively impact planetary health.^54,55^

**Table 3:** Application of Green Chemistry Principles and ABC Costing in Four Synthetic Pathways for rGO

Planning	Synthesis process strategy	Objectives	Green chemistry principle	Categories	Economic performance
	Common synthesis of rGO (16 h)	1. Green chemistry	#3, #4	Safety management, Sustainable design	ABC Method (#number of steps)
	Common synthesis of rGO (4 h)	2. Economic performance of the synthetic routes	#3, #4	Safety management, Sustainable design	ABC Method (#number of steps)
	rGO synthesis Guarana		#3, #4	Safety management, Sustainable design	ABC Method (#number of steps)
	rGO synthesis Biomass		#3, #4, #5 #7, #8,	Input selection and reduction, Safety management, Sustainable design	ABC Method (#number of steps)
Designing	Setting the problem into interconnecting tasks				
	Develop the tasks according to the objectives				

In nanotechnology, significant advances have focused on replacing hazardous reagents with safer alternatives such as plants, algae, and bacteria. rinciple #4: Safer Products: Utilizing molecular or intelligent design to prevent harmful interactions between products, living organisms, and the environment is a key strategy to minimize risks. This approach is particularly important for products with high market potential and large-scale production. Principle #5: Reducing the Use of Auxiliaries: This principle promotes minimizing solvent use during synthesis and purification, or selecting environmentally friendly alternatives when necessary. Aqueous media are preferred, and in nanotechnology, water-soluble precursors, centrifugation for purification, and green solvents like ionic liquids or exchangeable solvents support this approach.^56^ Principle #7: Use of Renewable Reagents: This principle is exemplified in biobased synthesis via nanobiotechnology, which harnesses the natural ability of biomolecules and renewable biological systems to reduce materials. This approach enables the production of diverse nanomaterials with unique biological, catalytic, and optical properties.^56^ Principle #8: Avoiding Derivatization and Minimizing Reaction Steps: Multistep processes and derivatization lead to higher consumption of chemical reagents and lower overall atomic efficiency. This principle advocates for avoiding energy-intensive techniques and the use of hazardous reaction auxiliaries.^56^

### 4.2 Activity-Based Costing (ABC) Method

To precisely calculate production costs, manufacturers have been encouraged to adopt effective strategies to enhance operational performance. The activity-based costing (ABC) approach has been embraced by various industries because it allows for a direct calculation of diverse costs, such as labor and resources, rather than relying on a single allocation factor. The ABC approach is an efficient method for estimating production costs, providing precise calculations. This method has been employed in decision-making across various research areas, including green manufacturing.^57^ The ABC method offers an alternative to common cost accounting systems by addressing their limitations. One of its key advantages is its ability to allocate overhead costs based on the direct costs associated with specific activities. As it can be shown in Figure 12, a two-stage procedure is crucial for allocating resources to cost objects. In the first stage, resources are assigned to activities based on resource drivers, which measure resource consumption. In the second stage, the resources allocated to each activity are treated as cost elements.

**Figure 12:**
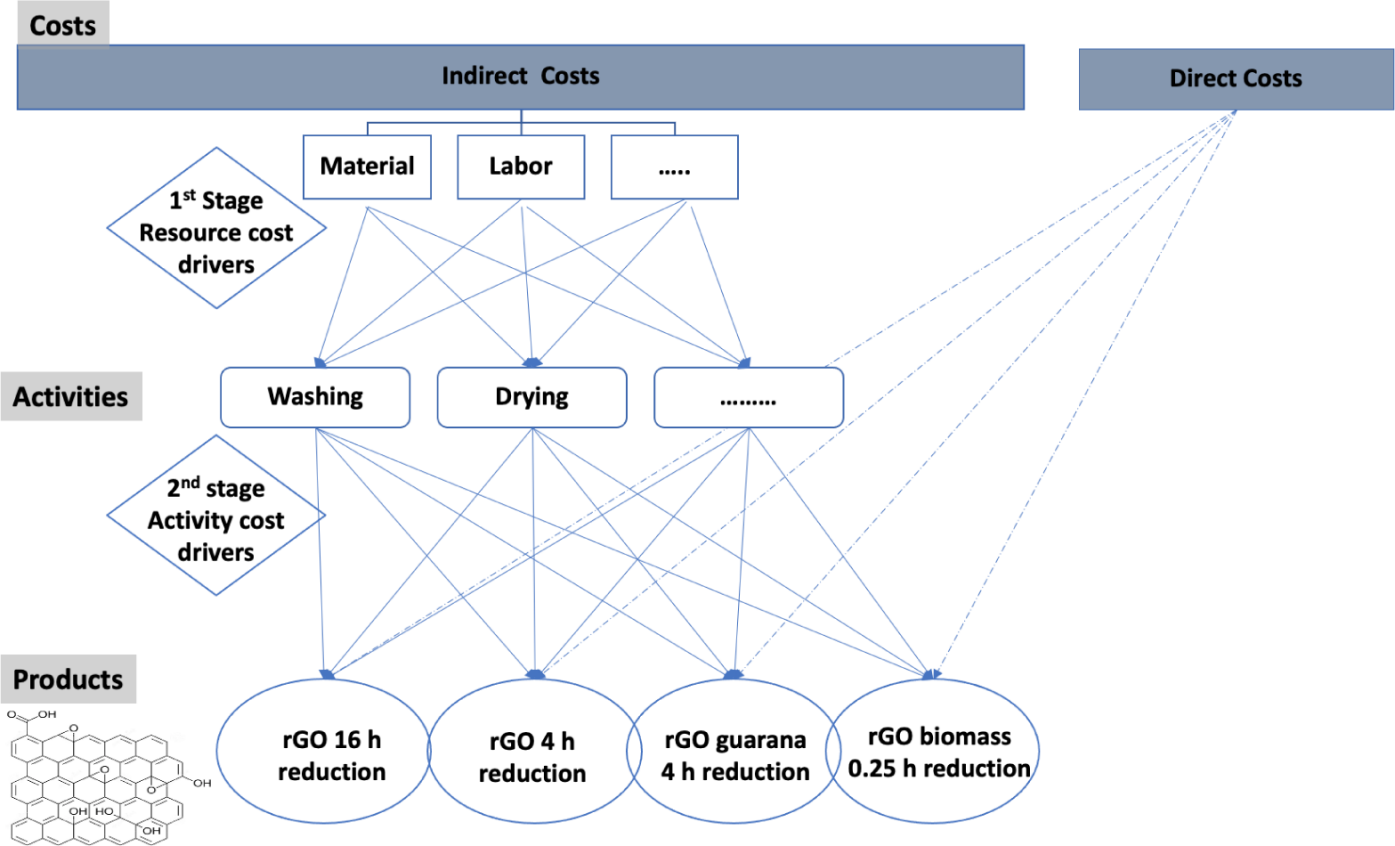
Cost assignment perspective of the Activity-Based Costing (ABC) method for rGO synthesis pathways.

The activity cost pool is a collection of cost elements, and the cost of an activity center or subprocess is formed by related activities or activity cost pools. In the second stage, cost elements for each activity are allocated to cost objects using activity drivers, which measure how much each cost object consumes the activities. Resource expenses assigned to an activity were determined by directly observing the time researchers spent on synthesis activities. Through a bottom-up analysis, each manufacturing process was divided into subprocesses, which were further broken down into various activities. Ultimately, to adhere to the ABC method guidelines, resources and the necessary resource and activity drivers for cost assignment were identified.

### 4.3 Costing Calculation Methodology

A cost model based on the ABC method was developed to compare the preparation and reduction processes in terms of synthesis costs, estimating the total synthesis cost at the activity level for both processes. This section includes a detailed explanation of all the equations used to calculate the total manufacturing cost, with the units of measurement for each element defined in Table 4. Before presenting the equations, several assumptions have been made to represent typical laboratory conditions: (a) a year has 227 working days, with each day consisting of 8 h; Furthermore, primary data collection, including equipment operating times and working hours, was conducted through direct observation.

**Table 4:** Overview of Costs and Their Formulas^a^

Cost Subcategory	Formula	Formula number	Ref
Raw material	$C_{R}=\underset{i=1}{\sum }\left(U_{i}\times N_{i}\right)$	(2)	^58^
Labor	$C_{L}=\sum \;w_{i}\times h_{i}\times n_{i}$	(3)	^58^
Energy	$C_{E}=P_{D}\times a\times t$	(4)	^59^,^60^
Maintenance	$C_{M}=u_{i}n_{i}+w_{i}h_{i}n_{i}$	(5)	^58^
Depreciation	$P=PV\times \frac{r}{1-{\left(1+r\right)}^{-n}}$	(6)	^58^

a *C_R_* is raw material cost in (€), *U_i_* = the price per unit for material *i* (€/unit), *N_i_* = the number of units for material *i* (number of units), *C_L_* is the total labor cost in (€), *W_i_* = the hourly wage of category *i* (€/hour per person), *h_i_* = the number of hours of category *i* (hours), *n_i_* = the number of employees of category *i* (number of people), *C_E_* is the cost of energy, *P_D_* = the nominal power of the apparatus (kW), *t* = the usage time of the apparatus (hours), where *a* is the Load Factor (0 < LF < 1), *C_M_* is the maintenance cost, *u_i_* = the price per unit for material *i* (€/unit), *n*_*i*_ = the amount of units for material *i* (number of units). *w*_*i*_ = the hourly wage of category *i* (€/hour per person), *h_i_* = the number of hours of category *i* (hours), *n_i_* = the number of employees of category *i* (number of people), *P* = Payment *PV* = Present Value, *r* = rate per period, *n* = number of periods, (€) = ∑(*w_i_* × *h_i_* × *n_i_*) hourly wage of category *i* × hours spent × number of employees, CRE = the cost of replacement of equipment (€) = *A* + *B* + *C* damage to equipment + damage to infrastructure + damage to raw materials, CHB = the cost of human benefits (€/year) = *C*_1_ × *n*_1_ + *C*_2_ × *n*_2_ *C*_1_, *C*_2_ = the cost of one light injured and serious injured worker (*D*/person); *n*_1_, *n*_2_ = the number of light injured and serious injured workers (# injured persons), CIB = the cost of insurance benefits (€/year) = *P* × Ip Current premium × expected increase in premium (%), COC = other costs, i.e., cleaning and root cause analysis (€/year) = ∑(*w_i_* × *h_i_* × *n_i_*), hourly wage of category *i* × hours spent × number of employees, CA = the yearly cost of accident in Euros (€), *h_i_* = the total number of hours of category *i* (hours), *H* = the total number of working hours in a year (1920).

### 4.4 Synthesis Cost Model

Considering the specifics of the ABC method and the previously stated assumptions, a series of mathematical equations were developed to estimate the total synthesis cost for the two examined processes. According to eq 1, the total synthesis cost (TCO) for each process includes components such as energy consumption (*C*_*E*_), material costs (*C*_*R*_), labor costs (*C*_*L*_), maintenance (*C*_*M*_), and equipment depreciation (*C*_*D*_). Cost profiling is based on the notion that each activity represents a source of cost, making detailed information about each step, cost, and activity crucial. The model consists of three tiers: input, calculations, and outcomes, with the outcomes presented in either numerical or graphical form.

4 $$\mathrm{TCO}=C_{R}+C_{E}+C_{L}+C_{M}+C_{D}$$

where

TCO is the total cost of ownership of the synthesis process

*C_R_*- Raw material cost

*C_E_* - Energy cost

*C_L_* - Labor cost

*C_M_*- Maintenance cost

*C_D_* - Depreciation of apparatus and equipment

All factors and formulas used for their calculations are presented in Table 4, which serves as the foundation of the model, clearly outlining which costs are considered and the methods used to calculate them.

Considering the specifics of the ABC method and the previously mentioned assumptions, several mathematical equations were developed to estimate the total cost of the synthesis processes being investigated.

### 4.5 Risk Return Frontier

By taking into account our previous research graphene is a moderate risk material (low cost – moderate toxicity) compared to other alternatives.^61,62^ To evaluate both the risks (i.e., complexity of the synthetic pathway) and returns (i.e., cost-efficiency of the synthesis process), for rGOs this study applies the Markowitz risk-return framework.^63,64^ The risk-return profiles for reduced graphene oxide (rGO), illustrated in Figure 9, are modeled using Markowitz’s graphical representation of risk-return curves, where (i) Risk corresponds to the complexity of the synthesis pathway, and (ii) Return represents the cost savings achieved during synthesis.

All materials are evaluated using the same production quantity to ensure comparability; however, this parameter can be adjusted to validate results across different scales. In this model, the expected synthesis cost of rGO is denoted as *c*_expect_. The synthetic pathway can either increase or reduce this cost. A complex pathway with *X* steps and probability p leads to higher expenses, calculated as Cost of complex pathway = *p* × *X* × *c*_expect_. In contrast, a simplified pathway reduces the synthesis cost by crGO, with the cost expressed as

Cost of simplified pathway = (1 – *p*) × *X* × crGO. Thus, the net gain (or return) from selecting a simpler pathway is Return = (1 – *p*) × *X* × crGO – *p* × *X* × *c*_expect_. As expected, increasing the number of steps (n) in the synthesis process tends to raise the probability (*p*) of encountering higher costs (cr*GO*), thereby affecting the overall risk-return profile

- (i) *x*-axis: Risk = *p* × *N* × crGO
- (ii) *y*-axis: Return = ((1 – *p*) × *N* × crGO – *p* × N *×* crGO

## 5 Conclusions and Key Challenges

This study integrates guarana and pomegranate into the field of inorganic synthesis, demonstrating their potential to simplify laboratory procedures while advancing environmentally sustainable practices. The proposed green synthesis of reduced graphene oxide (rGO) using pomegranate biomass offers notable advantages. SAXS analysis of the rGO synthesized via this biomass-based route reveals a nanostructure comparable to that of conventionally produced rGO materials. Guarana served effectively as a reducing agent, with its efficacy and stability confirmed during the process. Moreover, the use of pomegranate biomass significantly reduces costs by eliminating the need for multiple complex synthesis steps. Economic analysis highlights that the reduction stage is the primary cost driver in the rGO synthesis process. While labor and energy costs are important, the number of synthesis steps plays a critical role in determining the total cost of ownership (TCO). This analysis emphasizes the value of integrating experimental data with economic evaluation to inform decisions that reduce synthesis costs and support large-scale implementation. By bridging green chemistry principles with streamlined synthesis procedures, the approach effectively removes economic barriers and enhances the sustainability of rGO production. Unlike conventional methods that rely on expensive reagents and energy-intensive steps, this strategy offers a low-cost, high-yield, and environmentally friendly alternative. The combined green and economic benefits of this methodology establish it as a sustainable and efficient path forward, contributing to the advancement of eco-conscious practices in inorganic chemistry.

Numerous researchers have explored the fabrication of graphene-based hybrid nanocomposites to address the evolving demands of industrial research. Continued investigation in this area holds promise for the development of advanced materials with minimized environmental impact. For example, a study by Singh et al. demonstrated that incorporating rose-petal extracts uniformly into graphene oxide (GO) not only enhances its performance but also opens new avenues for applications in energy storage, electronics, and catalysis. This homogeneous dispersion likely improves the biocompatibility of GO, offering the potential for the tailored design of GO-based materials by integrating additional natural extracts or dopants for specific functionalities.^12^ Additionally, Abdullah et al. emphasized the utility of graphene-based materials in modifying metal oxides. Graphene enhances the adsorption capacity of metal oxides for organic pollutants while also reducing electron–hole recombination, thereby improving photocatalytic efficiency. In particular, reduced graphene oxide (rGO) demonstrated notable photocatalytic performance in the degradation of methylene blue (MO) dye under visible light irradiation, highlighting its promise as an effective nanocatalyst.^10^

Nanographene has emerged as a highly promising platform for biomedical applications, prompting an urgent need for collaborative efforts across the disciplines of physics, chemistry, biology, and medicine to fully realize its potential. The transformation from graphene oxide (GO) to reduced graphene oxide (rGO), and ultimately to pristine graphene, especially when combined with metal oxides, yields composite materials with exceptional properties. These composites show great promise for the scalable production of specialized compounds and cost-effective biosensors, with applications spanning environmental monitoring, clinical diagnostics, security, and pharmaceutical research.^6^ Carbon nanomaterials (CNMs) such as graphene, carbon nanotubes (CNTs), and their derivatives possess numerous adsorption sites, low density, adjustable electrical properties, high carrier mobility, low operating temperatures, long lifespans, and ease of recovery. These characteristics make them ideal sensing materials for detecting a wide range of toxic pollutant gases and volatile organic compounds (VOCs). Many studies have reported enhanced sensitivity in these materials, enabling the detection of harmful VOCs at parts-per-billion (ppb) levels.^4^ The synergistic interaction between bimetallic metal oxides (bi-MO) and 2D reduced graphene oxide (rGO) nanosheets enhances their electronic properties in photochemical and electrochemical settings, enabling effective applications in environmental and biological fields.^5^

A new synthetic route has been developed to streamline the production of rGO, reducing the number of synthetic steps involved. This synthesis process is influenced by technical, environmental, and economic tools used to evaluate sustainability. Economic considerations can significantly impact the technical aspects of material design. The intersection of sustainability and economic pressures is vital for adopting more sustainable synthesis processes, and this relationship can be synergistic if both economic and environmental factors are integrated into the synthesis design process. From a green chemistry standpoint, “green” nanomaterials are structures engineered to minimize environmental and public health risks during their design, synthesis, application, and disposal. Practically and scientifically, “green” nanomaterials are those produced in a way that leads to a lower ecological impact. In the short term, chemical processes can be improved by replacing toxic solvents with safer common alternatives and adopting process intensification. However, for substantial progress, future chemical processes must prioritize reducing energy consumption and employing renewable materials.

## Supplementary Material



## Notes

Brief descriptions in nonsentence format listing the contents of the files supplied as Supporting Information; in particular, the cost of a process step to assess the efficiency of various synthetic pathways is described in details (PDF)
