# Supplementary material for: Various Reduced Graphene Oxide Green Synthetic Routes: Comparing the Cost Procedures
Source: ACS Omega. 2025 Aug 4;10(32):36221–37. doi: 10.1021/acsomega.5c04090 (PMC12368663; doi:10.1021/acsomega.5c04090)
Supplement: Supplementary file 1 [file ao5c04090_si_001.pdf]

Various reduced graphene oxide green synthetic routes: Comparing the cost procedures

Despina A. Gkika[1,*], Konstantinos N. Maroulas[1], George Z. Kyzas[1,*]
[1]*Hephaestus Laboratory, School of Chemistry, Faculty of Sciences, Democritus University of Thrace, GR-65404 Kavala, Greece*

*Corresponding authors: kyzas@chem.duth.gr (G.Z.K.); despinagkika@gmail.com (D.A.G.), *Hephaestus Laboratory, School of Chemistry, Faculty of Sciences, Democritus University of Thrace, GR 65404 Kavala, Greece, Tel.: +30-2510-46-2218*

Each synthesis process comprises various subprocesses, as illustrated in **Figure S1**.

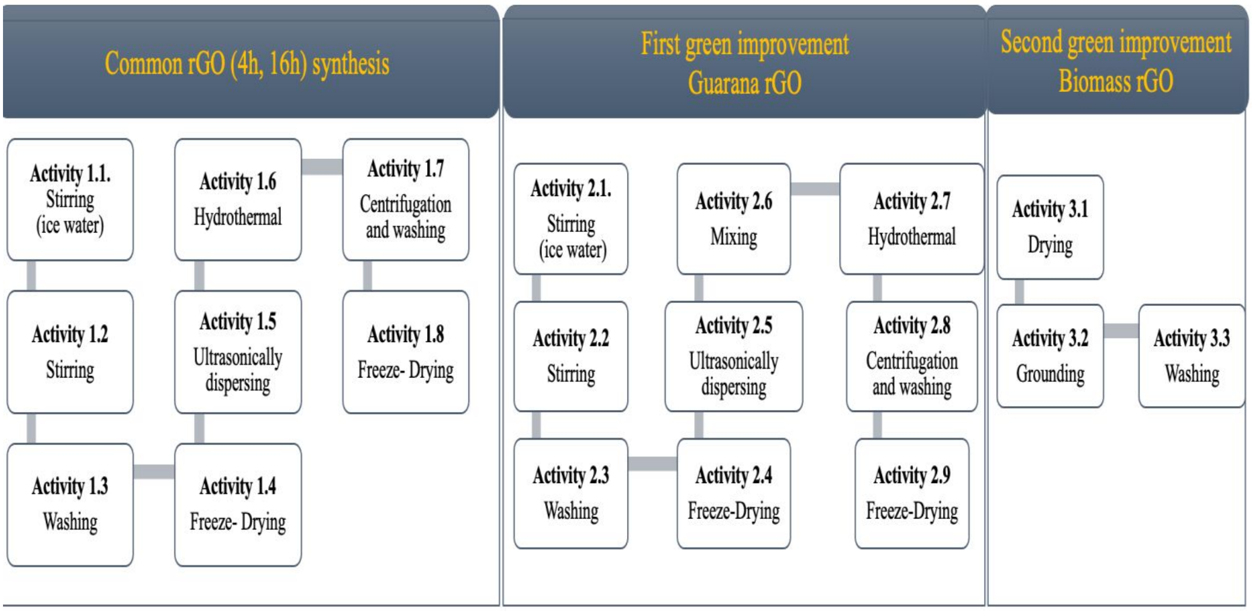

Figure S1. Flow diagrams illustrating the various synthetic pathways for the rGO components.

The common synthesis process (16 or 4 h) involves eight distinct activities, while the guarana synthesis process includes nine activities, and the biomass process consists of three activities.

SI.1. Raw material cost

Tretyak & Sloev suggest the virtual integration of clients into business process innovation through an interactive approach, enabling the creation and distribution of value [58]. Consequently, the primary goal during this phase was to investigate the market prices for the materials and printers under study, as well as to explore the commercial catalog offerings of each vendor [59].

$$C_R = \sum_{i=1}(U_i \times N_i) \quad \textbf{(2)}$$

SI.2 . Labor cost [59].

Before calculating labor costs, certain assumptions regarding standard laboratory conditions are established: a full working year consists of 250 days, with each day comprising 8 working hours. The synthesis process for each type of rGO requires one experienced researcher, but only a fraction of that time involves active labor: 0.3 actual person-hours are needed for rGO 4h (despite a total process duration of 4 hours), 0.3 person-hours for rGO 16h (with a 16-hour total duration), 0.3 person-hours for rGO guarana (4-hour process), and 0.016 person-hours for rGO biomass (0.5-hour process). Labor costs were based on average salary data sourced from Glassdoor [60] chosen for its comprehensive database that includes position- and country-specific wage estimates, along with details on sample size, wage ranges, and calculation methodology. According to this source, the average hourly wage for an experienced researcher in Greece is approximately 4.68€.

$$C_L = \sum w_i \times h_i \times n_i \quad \textbf{(3)}$$

SI.3. Energy cost

Energy consumption was estimated following the method proposed by [61], which focuses on breaking down the energy usage of each piece of equipment used in the studied activities. This method involves three steps, beginning with identifying the necessary equipment and assessing their nominal power. This information is typically available in the technical documentation provided by the equipment manufacturers. Although nominal power is higher than the actual energy used during the process, it serves as a reference point when more precise measurements are not feasible. The final step involves estimating the duration of time each piece of equipment was in operation [62]. Once the relevant data is collected, the actual energy consumption can be calculated using the following formula:

$$C_E = P_D \times t \quad \textbf{(4)}$$

where

C_E is the cost of energy,

P_D =the nominal power of the apparatus (kW),

t = the usage time of the apparatus (hours).

The final result should take into account the load factor.

$$C_E = P_D \times a \times t \quad \textbf{(4.1)}$$

where a is the Load Factor (0 <LF < 1)

Given that the required time was specified, the only remaining variable to assess was the load factor. The cost was calculated by multiplying the amount of energy consumed in KWh by the price per KWh in Euros (Greece). This price reflects the average rate for 2025, as per EU data, and is sourced from the Greek Public Power Corporation [63]. With this data, the incorporation of the energy dimension into the model became feasible.

SI.4. Maintenance cost

Worn-out materials need to be replaced during the maintenance of lab equipment [59]. Maintenance costs encompass not only the potential replacement costs but also the expenses associated with employee activities throughout the process.

$$C_M = u_i n_i + w_i h_i n_i \quad \textbf{(5)}$$

SI.5. Depreciation cost

Depreciated cost refers to the value of an asset after accounting for depreciation. It represents the portion of the asset that has not yet been fully consumed. The annuity method is commonly used to simplify calculations of depreciation. This method is applicable when annual net operating revenue is stable, and the replacement cost of the equipment remains constant [59]. As a result, the total cost includes both interest and depreciation. At the end of each year, the equipment's value will correspond to the discounted present value of the remaining annuity payments, while the depreciation cost will be equivalent to these values.

$$P = PV \times \frac{r}{1-(1+r)^{-n}} \quad (6)$$

The annuity factor facilitates quick calculations when determining the payment for an annuity with a known present value. This can be done by referencing a table to find the factor for a specific interest rate and time period. For the investment cost annuities, a discount rate of 3% was applied.

	**Material cost (€)**	**Labor cost (€)**	**Energy cost (€)**	**Maintenance cost (€)**	**Depreciation cost (€)**	**Total after tax and discount (€)**	**Cost per gr (before tax and discount) (€)**
GO	53.41	24.61	6.74	0.40	1.92	104.74	15.89
rGO 4h	4.80	1.41	6.58	1.48	13.04	34.27	170.15
rGO 16h	4.80	1.41	9.65	1.68	13.04	38.20	232.75
rGO Guarana 4h	5.24	1.41	6.58	1.48	13.04	34.81	30.91
rGO Biomass reduction	4.44	0.08	0.01	0.00	0.16	5.64	15.45
rGO Biomass preparation	-	-	0.06	0.00	1.16	1.47	4.03
rGO Biomass total	4.44	0.08	0.07	0.01	1.31	7.11	19.48

**Material**			
**Synthesis Process**	**Preparation**	**Reduction**	**Percentage**
rGO 4h	53.41	5.24	-90%
rGO 16h	53.41	4.80	-91%
rGO Guarana 4h	53.41	4.80	-91%
rGo Biomass+Ferrocene	0.00	4.44	100%

**Energy**			
**Synthesis Process**	**Preparation**	**Reduction**	**Percentage**
rGO 4h	6.74	6.58	-2%
rGO 16h	6.74	9.65	43%
rGO Guarana 4h	6.74	6.58	-2%
rGo Biomass+Ferrocene	0.06	0.01	-85%

**Maintenance**			
**Synthesis Process**	**Preparation**	**Reduction**	**Percentage**
rGO 4h	0.40	1.48	271%
rGO 16h	0.40	1.68	321%
rGO Guarana 4h	0.40	1.48	271%
rGo Biomass+Ferrocene	0.00	0.00	-17%

**Depreciation**			
**Synthesis Process**	**Preparation**	**Reduction**	**Percentage**
rGO 4h	1.92	13.04	578%
rGO 16h	1.92	13.04	578%
rGO Guarana 4h	1.92	13.04	578%
rGo Biomass+Ferrocene	1.16	0.16	-86%

**Total cost (per g)**			
**Synthesis Process**	**Preparation**	**Reduction**	**Percentage**
rGO 4h	15.89	170.15	971%
rGO 16h	15.89	232.75	1365%
rGO Guarana 4h	15.89	30.91	95%
rGo Biomass+Ferrocene	4.03	15.45	283%

**Process duration (hours)**			
**Synthesis Process**	**Preparation**	**Reduction**	**Percentage**
rGO 4h	41.25	4.00	-90%
rGO 16h	41.25	16.00	-61%

rGO Guarana 4h	41.25	4.00	-90%
rGo Biomass+Ferrocene	2.00	0.25	-88%
